# On opportunity for emergency cesarean hysterectomy and pregnancy outcomes of patients with placenta accreta

**DOI:** 10.1097/MD.0000000000007930

**Published:** 2017-09-29

**Authors:** Xiang Kong, Yan Kong, Jin Yan, Jin-Ju Hu, Fang-Fang Wang, Lei Zhang

**Affiliations:** a Department of Obstetrics and Gynecology, Clinical Medical College of Yangzhou University; b Department of Obstetrics and Gynecology, The Women and Children Hospital of Yangzhou, Yangzhou, Jiangsu, China.

**Keywords:** cesarean hysterectomy, emergency cesarean section, maternal and neonatal outcomes, placenta accreta, scheduled cesarean section

## Abstract

Effective diagnosis and clinical management of placenta accreta (PA) in China are not clear. The purpose of the study was to analyze the risk factors and diagnosis of PA, maternal and neonatal outcomes in patients with PA. It was a retrospective study of cases with PA, confirmed by histologically and/or clinically suspected during 3 years in 2 tertiary referral hospitals. The incidence rate of patients with PA, who had history of artificial abortion, cesarean section (CS), and placenta previa (PP) was 94%, 70%, and 72%, respectively. In 29 patients of scheduled CS group, 12 cases were performed with cesarean hysterectomy. Mean estimated blood loss (EBL) was 1.5 L, and 17 babies were admitted to neonatal intensive care unit (NICU). In the 18 cases of emergency CS group, 6 cases were performed cesarean hysterectomy. Mean EBL was 2.4 L, and 16 babies were admitted to NICU. The difference of mean EBL, cases of fetal admitted to intensive care unit in 2 groups was significant difference (*P* < .05).

Women with history of uterine curettage, CS or PP are more likely to have PA. PA should be diagnosed early and accurately via ultrasound and magnetic resonance imaging. Maternal and neonatal outcomes in the scheduled CS are better than in emergency CS. Emergency peripartum hysterectomy is a feasible method under the circumstances of heave, fast bleeding, and the failure of conservative surgery.

## 1 Introduction

Placenta accreta (PA) is rare, but associated with many severe complications, such as intractable postpartum hemorrhage, cesarean hysterectomy, resultant admission to intensive care unit (ICU) or with neonatal morbidity including low birth weight and admission to neonatal intensive care unit (NICU).^[1–3]^ The risk factors and optimal management of PA are not very clear. The incidence of PA has been reported increased recently worldwide.^[4]^ In recent years, PA in China also occurred frequently, and this has been attributed to the rising rate of caesarean section, especially after Chinese Government changed the policy of family planning, which will lead to the result that women may have more children.^[4–6]^ However, reasonable and effective diagnosis, and clinical management of PA in China are not clear, especially the time of emergency cesarean hysterectomy.^[7,8]^ PA may cause severe complications, such as heavy blood loss and maternal death. Up to now, early diagnosis and effective clinical management still remain unpredictable.^[9–13]^ The purpose of this study was to analyze the risk factors of PA as well as to evaluate the time to perform emergency cesarean hysterectomy and pregnancy outcomes of scheduled and emergency cesarean section (CS) patients with PA.

## 2 Patients and methods

### 2.1 Patients and group

This retrospective cohort study was conducted at 2 tertiary referral hospitals: Clinical Medical College of Yangzhou University and the Women and Children Hospital of Yangzhou. The study was approved by ethics committees in the 2 hospitals. Women with PA, placenta previa (PP), and cesarean hysterectomy were confirmed by using the ICD-9 codes during the period from December 1, 2011 to November 30, 2014. The results of laboratory investigation and histopathology were obtained from the databases of the 2 hospitals. The general information such as patients’ age, gravidity, parity, gestational age in time of CS, etc., was gathered. The data also included estimated blood loss (EBL) during operation, and the unit of packed red blood cells (PRBC) transfusion was also given. In this study, patients were divided into 2 groups: scheduled CS and emergency CS. For the former group, it was planned to perform CS at least 1 day in advance; the latter was performed CS immediately in case of maternal hemorrhage or fetal distress.

Oxytocin was used after delivery when patients with PA were performed CS. Hemabate and misoprostol were applied when patients encountered uterine inertia. If the placenta could not strip or uterus had active bleeding after the placenta stripped, conservative surgeries were adopted such as local oppression and suture, B-Lynch suture, pelvic vascular ligation. When bleeding could not stop with these methods or blood bleeding quickly or EBL was >1500 mL in a short time, doctors communicated immediately with the family members and suggested to perform emergency hysterectomy.

### 2.2 Statistical analysis

SPSS 17.0 was used to analyze the data. Descriptive statistics’ methods were used to describe the cohort. Outcomes in women undergoing different management schemes were compared using Student *t* test, chi-squared analysis as appropriate. If *P* < .05, statistical significance existed. The statistical significance of the differences in continuous variables was assessed by 1-way analysis of variance. Chi-squared test or Fisher exact test was applied to determine the statistical differences among the groups in categorical variables.

## 3 Results

The total labor number was 29,220 in the 2 hospitals during 3 years. The cases of PP was 318 (10.9/1000) and the number of CS was 14,529 (49.7%). Forty-seven patients were diagnosed with PA, and the incidence of PA was 1.6/1000. Patients’ characteristics were shown in Table 1. Of the various risk factors, 44 (91%) patients had prior uterine curettage, 33 (70%) patients had multiple CS, 34 (72%) patients suffered from PP, and 28 (60%) patients had both the history of CS and PP. All patients received antenatal ultrasound more than once. Twenty-one (45%) patients were confirmed with PA via ultrasound. The patients did not receive magnetic resonance imaging (MRI) routinely in the 2 hospitals. Of the 47 patients, 8 patients were examined with MRI and only 4 (50%) patients were confirmed with PA. Forty-seven patients of PA were divided into scheduled CS group and emergency CS group. Patients from scheduled CS group with previous uterine curettage took up 27 (93%) and patients from emergency CS group had previous uterine curettage, 17 (94%). Patients from scheduled CS group with a history of previous CS took up 20 (68%) and patients from emergency CS group, 13 (72%). Patients from scheduled CS group with a history of PP took up 19 (66%) and patients from emergency CS group, 15 (83%). Patients from scheduled CS group with positive diagnosis via ultrasound took up 12 (41%) and patients from emergency CS group, 9 (50%).

**Table 1:**
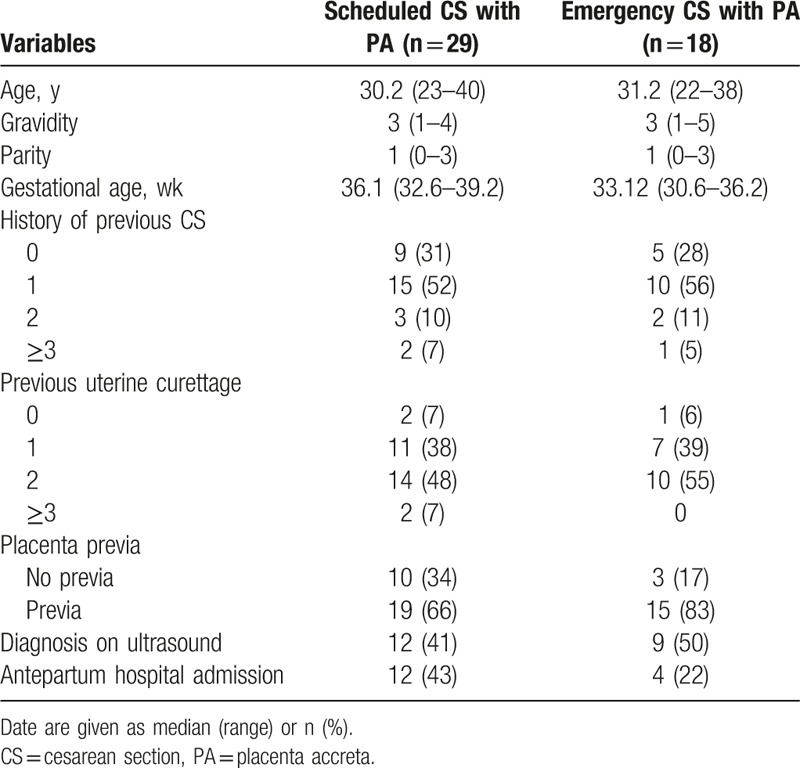
Patient characteristics.

Perinatal maternal outcomes of women with PA were summarized in Table 2. Twenty-nine patients with PA received scheduled CS. Placenta was removed from 20 (69%) patients. Due to massive blood loss and failure of conservative surgery, 12 (41%) patients received cesarean hysterectomy immediately. Eighteen cases with PA received emergency CS. Placenta was removed in 14 (78%) cases. When heavy bleeding occurred or conservative surgery failed, 6 (33%) cases received cesarean hysterectomy after discussing and communicating with the family members of the patients in time. Mean EBL in scheduled CS group was less than in emergency CS group. The rate of ICU admission in scheduled CS group was lower than in emergency CS group.

**Table 2:**
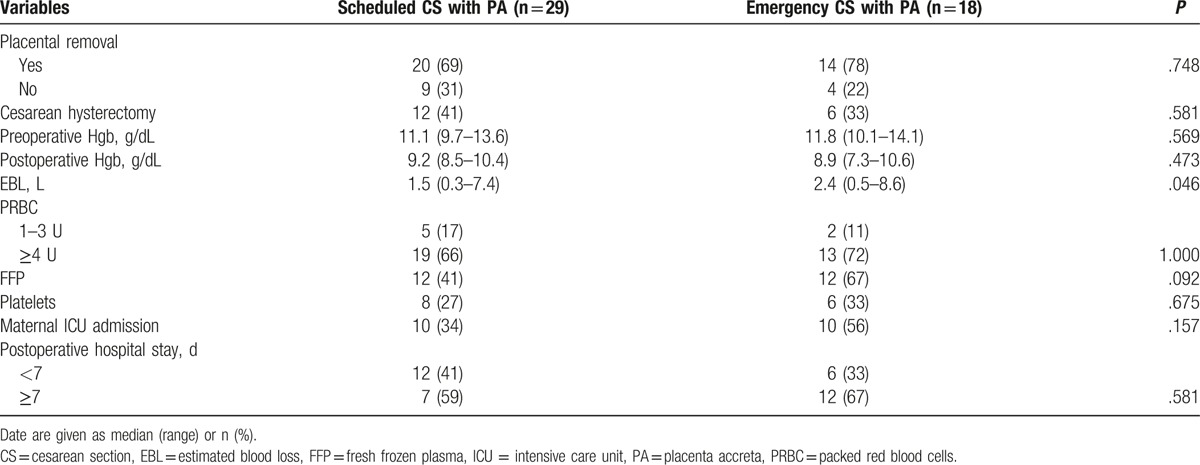
Comparison of maternal outcome with PA for scheduled cesarean section and emergency cesarean section (n = 47).

Neonatal outcomes were shown in Table 3. In scheduled CS group, more than half of babies were born between 34 to 37 weeks, similar to that in emergency CS group. But the percentage of the gestational age of babies was more than 37 weeks in scheduled CS group, higher than that in emergency CS group. Seventeen (59%) babies in scheduled CS group were admitted to NICU due to low birth weight. Sixteen (89%) babies in emergency CS group were admitted to NICU due to low birth weight and/or low Apgar score.

**Table 3:**
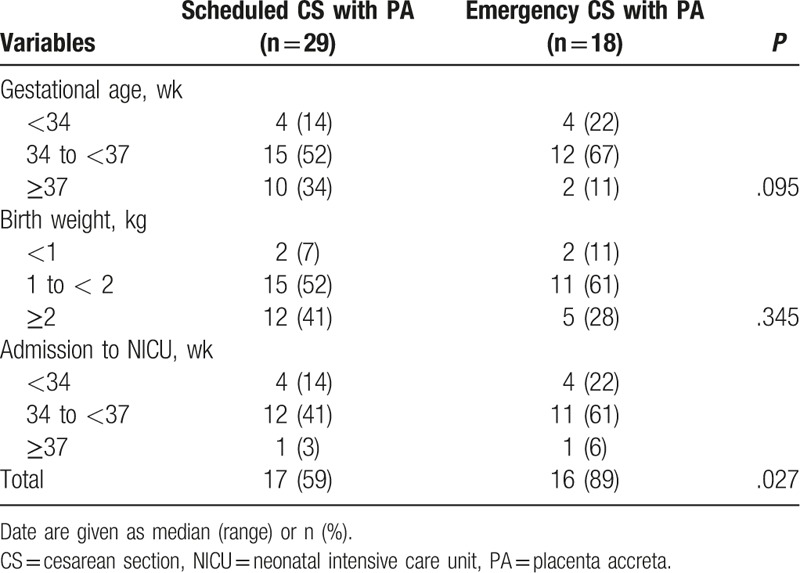
Neonatal outcome of women with PA.

## 4 Discussion

PA refers to abnormally adherent tissue to the uterus without easy separation, including PA, placenta increta, placenta percreta.^[2,9]^ When the placenta invades into the myometrium, it is defined as placenta increta; placenta percreta refers to placenta villi's invasion through the myometrium and into the serosa. PA takes up approximately 0.9% of all pregnancies. The incidence of PA is about 3 per 1000 deliveries worldwide, which has been a significant increase in incidence of PA over the past decade.^[1,9–11]^ Eller et al^[2]^ found that the incidence of PA in 2 tertiary care centers increased 120% in 12-year interval, from 5.4/10,000 deliveries in the first half of the study to 11.9/10,000 deliveries in the second half of the study. Women who had one or more prior cesarean deliveries and were identified with PP in the current pregnancy are more likely to have PA.^[14]^ Other risk factors of PA include previous uterine surgery, maternal age, or previous uterine curettage. In this study, the incidence of PA was 1.6/1000 deliveries. The result showed that high risk factors include previous uterine curettage (91%), especially repeated curettage. The second and third risk factors were prior cesarean delivery and PP. They may occur to women who usually chose artificial abortion after they failed in contraception. Because of the previous policy of family planning in China, most women had the experience of artificial abortion, which is the first factor to cause PA. The rate of CS reached 50%, and in some areas the rate was even higher than 80% in China.^[15–17]^ Chinese Government issued a new family planning policy that allows the husband and wife to have 2 children. This policy will come into effect from 2015. Consequently, the change of the policy will lead to the increase of deliveries. Although many risk factors have been described above, the actual cause of the increased incidence of PA still remains unknown.^[9,13]^ Complications related to PA have also increased in the past decade. Both PP and PA are associated with the history and frequency of prior CS.^[15]^

The diagnosis of PA included pathologic specimen after operation and the use of sonography and MRI during antenatal period.^[10]^ Preoperative diagnosis is of paramount importance. It improves effective management planning and reduces morbidity. Although more studies have attempted to address early diagnosis of PA, the application of ultrasound, and MRI yielded different results.^[18,19]^ PA should be suspected in women who have PP, a history of CS, and other uterine surgery. Many studies have shown the efficacy of sonography in the diagnosis of PA. Dwyer et al^[10]^ found that ultrasound had sensitivity of 93% and specificity of 71% in diagnosis of PA. But in our study, the sensitivity of sonography had only 45% (21/47) in diagnosis of PA, lower than that obtained through other researches. There were mainly 2 reasons for this. First, the incidence of PA was still low; second, lack of experienced sonography practitioner. This problem can be overcome by training doctors in ultrasonographic department. Similar diagnostic accuracy of sonography and MRI for prenatal diagnosis of PA has been found.^[18,19]^ But due to the higher cost of MRI and stringent technical requirement to the doctors, it is usually not the first choice in China. In special cases, MRI may be used in combination with sonography to improve prenatal diagnosis of PA. Antenatal diagnosis is a key factor in optimizing the maternal and neonatal outcome.

PA is a severe clinical problem as patients are at the risk of massive hemorrhage and preterm delivery. It has become one of the most common indications for emergency peripartum hysterectomy. Choosing suitable operation method and time are very important. PA causing emergency peripartum hysterectomy accounts for 45% to 73.3%.^[20]^ Doctors should conduct hysterectomy immediately when they met these situations. One situation is that a wide range of PA adherent to bladder, which causes the placenta inseparable; another situation is that patients with a history of CS plus complete PP and placenta implantation in lower uterine segment and cervical muscle layer. If conservative surgical treatments fail and bleeding is difficult to control, hysterectomy should be used. In this study, hysterectomy was conducted immediately when blood loss was more than 1.5 L and/or bleeding was fast. In scheduled CS group, we communicated with the families to decide the surgery method before operation, and we did not insist on removing placenta before turning to cesarean hysterectomy during operation. But in emergency CS group, when meeting PA during operation, we explained the situation to the family members of the patients, who usually hoped to remove placenta and reserve uterus except patient's situation threatening to the patient's life. This is understandable according to Chinese tradition. Research findings showed that it is not an ideal choice to remove placenta because removal of placenta would cause many complications to patients. These findings were also supported by other relevant researches.^[21]^

In this study, although the rate of cesarean hysterectomy in the 2 groups is similar, incidence of maternal complications such as EBL, transfusion volume of PRBC, fresh frozen plasma, and maternal ICU admission in emergency CS group was higher than in scheduled CS group. It is better for patients with PA under selectable condition instead of emergency without adequate preparation to operate. Therefore, scheduled delivery close to 37 weeks of gestation time may be reasonable. If patient's situation is stable, delivery by CS should be taken into consideration after 37 weeks. Al-Khan et al^[1]^ compared scheduled peripartum hysterectomy with emergency peripartum hysterectomy and found that patients in the emergency hysterectomy group had greater intraoperative blood loss than those who had scheduled hysterectomy. Prevention of complications requires a multidisciplinary team cooperation and careful preparation for delivery. The patient and her family members should be counseled preoperatively about the significance of hysterectomy and the likely need of transfusion of blood and blood products. Although scheduled delivery should be considered as the best choice, contingent plans should be made for possible emergency situation.^[1,2,22]^ Many disciplines including maternal-fetal medicine, anesthesiology, vascular surgery, and urology should cooperate together so as to reduce complications of mother and fetus.^[23,24]^

At present, the relationship between neonatal outcomes and PA is unclear.^[1,2,15,25]^ Neonatal morbidity in our study was also significantly high. Neonatal morbidity was more serious before 34 weeks of gestational age, and all of them were transferred to NICU. Compared with the emergency CS, scheduled CS had advantages in many aspects in terms of gestational weeks of birth before 37 weeks, neonatal weight below 2 kg, and NICU admission. Therefore, scheduled CS until 37 weeks could reduce neonatal morbidity. More data are required before selecting the optimal timing of delivery in women with PA.

In conclusion, considering the increased rate of CS and risk of maternal morbidity, PA should be diagnosed early and accurately via ultrasound and MRI, especially in those with risk factors. Maternal and neonatal outcomes in the scheduled CS are better than in emergency CS. Emergency peripartum hysterectomy is a feasible method under the circumstances of heave, fast bleeding, and the failure of conservative surgery. Maternal and neonatal outcomes of patients with PA should be studied in multicenter so as to obtain more clinical experience in diagnosis and management.

## Acknowledgments

The authors thank Dr. Dan Lu (Head of Obstetrics at Department of Obstetrics and Gynecology, Clinical Medical College of Yangzhou University) and Dr. Yixiong Wang (Vice Director at the Women and Children Hospital of Yangzhou), for their sincere assistance in this research.
